# Development and Implementation of an Innovative Framework for Automated Radiomics Analysis in Neuroimaging

**DOI:** 10.3390/jimaging10040096

**Published:** 2024-04-22

**Authors:** Chiara Camastra, Giovanni Pasini, Alessandro Stefano, Giorgio Russo, Basilio Vescio, Fabiano Bini, Franco Marinozzi, Antonio Augimeri

**Affiliations:** 1 Department of Mechanical and Aerospace Engineering, Sapienza University of Rome, Eudossiana 18, 00184 Rome, Italy; giovanni.pasini@uniroma1.it (G.P.); fabiano.bini@uniroma1.it (F.B.); franco.marinozzi@uniroma1.it (F.M.); 2 Institute of Molecular Bioimaging and Physiology, National Research Council (IBFM-CNR), 90015 Cefalù and 88100 Catanzaro, Italy; alessandro.stefano@ibfm.cnr.it (A.S.); giorgio-russo@cnr.it (G.R.); or basilio.vescio@biotecnomed.it (B.V.); 3 Biotecnomed SCARL, Campus Universitario di Germaneto, Viale Europa, 88100 Catanzaro, Italy; antonio.augimeri@biotecnomed.it

**Keywords:** radiomics, software package, machine learning, image analysis, neuroimaging

## Abstract

Radiomics represents an innovative approach to medical image analysis, enabling comprehensive quantitative evaluation of radiological images through advanced image processing and Machine or Deep Learning algorithms. This technique uncovers intricate data patterns beyond human visual detection. Traditionally, executing a radiomic pipeline involves multiple standardized phases across several software platforms. This could represent a limit that was overcome thanks to the development of the matRadiomics application. MatRadiomics, a freely available, IBSI-compliant tool, features its intuitive Graphical User Interface (GUI), facilitating the entire radiomics workflow from DICOM image importation to segmentation, feature selection and extraction, and Machine Learning model construction. In this project, an extension of matRadiomics was developed to support the importation of brain MRI images and segmentations in NIfTI format, thus extending its applicability to neuroimaging. This enhancement allows for the seamless execution of radiomic pipelines within matRadiomics, offering substantial advantages to the realm of neuroimaging.

## 1. Introduction

In recent decades, medicine has witnessed a significant digitization of information generated during routine clinical practice, leading to an increase in the development of software tools to analyze them [1].

Simultaneously, Artificial Intelligence (AI) applied to medical research, particularly through Machine and Deep Learning algorithms, has streamlined the processing of “Big Data” [2], referring to large volumes of data that require advanced technologies and techniques for acquisition, storage, distribution, management, and analysis.

One noteworthy innovation in this field is radiomics, an emerging discipline leveraging AI techniques to revolutionize the interpretation of digital radiological images [3].

Radiomics allows these images to transcend mere anatomical representations, potentially providing insights into several physiological and pathological processes.

The primary objective of radiomics is to extract quantitative data from radiological images, which are subsequently processed using appropriate data analysis methods.

This approach enables clinicians to glean more accurate diagnoses and provides valuable information such as predicting treatment response or disease progression [4].

Therefore, radiomics is paving the way for personalized medicine, promising improved quality of life, increased survival rates, and reduced healthcare costs [5].

A typical radiomic pipeline entails standardized procedures for extracting, analyzing, and interpreting quantitative features from medical imaging data.

The first step involves image acquisition via examinations like CT scans, MRI, or PET scans, often managed through the PACS (Picture Archiving and Communication System) [4] and employing DICOM (Digital Imaging and Communications in Medicine) [5] standards for communication.

Subsequently, segmentation identifies and delineates regions of interest (ROI), creating a binary mask where pixels with a value of 1 belong to the target, while pixels with a value of 0 do not.

Feature extraction follows preprocessing and filtering, aiming to reduce noise and highlight characteristics like contours. The extracted features are categorized as follows:

1. Shape and morphological features: describing the size and shape of the ROI independently of grayscale intensity distribution;
2. First-order statistical features: describing voxel intensity distribution within the masked image region;
3. Texture features: capturing information about grey-level patterns within the VOI (Volume Of Interest).

Tools like PyRadiomics [6], CERR (Computational Environment for Radiotherapy Research) [7], or LIFEx [8] aid in accurate feature extraction.

Once features are obtained, the next step is feature selection, often accomplished through the use of Machine Learning algorithms. Python-based libraries like SciKit-learn [9] or TensorFlow [10] are widely used in this context.

Finally, the workflow concludes with the construction of a predictive model, utilizing the selected features to derive meaningful insights. Predictive modeling encompasses methodologies and techniques capable of making predictions about future data or events based on available data. Generally, in order to construct a predictive model, Machine Learning models such as Support Vector Machine (SVM) [11], K-nearest Neighbors (KNN) [12], and Linear Discriminant Analysis (LDA) [13] are employed, while cross-validation is used for evaluating its performance.

Although using several software tools to complete a radiomic analysis may not represent an absolute problem, having a single interface guiding the entire pipeline could be advantageous in the clinical context. This limitation was overcome through the development of the matRadiomics application [14].

The primary goal of matRadiomics is to integrate the entire radiomics workflow into a single environment, enabling users to complete the workflow seamlessly without the need to switch between different software tools. By using this application, users can import and inspect biomedical images, identify and segment a target, extract desired features, select features, and build predictive models using Machine Learning algorithms [15]. It also focuses on result reproducibility by tracking matRadiomics configuration options set by the user through metadata.

To date, the application has been primarily designed and utilized to perform radiomic workflows on DICOM images related to tumor lesions [16]. While traditional approaches often face limitations in capturing the full spectrum of tumor characteristics, leading to potential misclassifications and impacting therapeutic decisions, radiomics provides a comprehensive and detailed characterization of the tumor microenvironment, emerging as a promising solution to address these challenges [17].

In neuroimaging, radiomics is becoming increasingly crucial to highlight specific brain characteristics through the identification of possible biomarkers [18]. Leading the entire radiomic analysis within a single software could represent a significant advancement for both clinicians and researchers.

The aim of this work is to extend the application of the functionalities to include its use in the context of neuroimaging as well. We included a new module, *NIfTIModule*, which allows users to import and process brain MRI images and segmentations in NIfTI format. Additionally, the *classificationModule* was expanded to incorporate new predictive models, including SVM and Random Forest.

To evaluate the reliability and robustness of the matRadiomics extension, we conducted a case study using openly available data from the Parkinson’s Progression Markers Initiative (PPMI) dataset. We utilized matRadiomics to extract radiomic features from the MRI images and segmentations, followed by the construction of predictive models using both SVM and Random Forest algorithms. The performance of the models was assessed using accuracy, precision, and recall metrics.

The source code and the documentation are available upon request to the authors.

## 2. Materials and Methods

### 2.1. Architecture

MatRadiomics is designed with a modular architecture that seamlessly integrates both Matlab and Python environments, harnessing their respective strengths for comprehensive functionality.

MatRadiomics backend was coded in MATLAB 2021b [19], which manages its functionality, while the graphical interface was created using the Matlab App Designer extension, an interactive development environment for designing app layouts and programming their behavior.

The application integrates a complete Python environment to manage the dependencies required for its functioning. Furthermore, matRadiomics contains ad hoc Python modules that can be invoked using the *py.* syntax. This syntax allows Matlab to call either built-in Python functions or functions implemented in these ad hoc Python modules.

This tool interacts with several core libraries: (i) the Pydicom v2.2.2 [20] library interfaces with the DICOM standard to read metadata associated with medical images; (ii) PyRadiomics v3.0.1 [8], an open-source package for extracting radiomic features from medical images; and (iii) SciKit-learn v1.0.1 [11], an open-source Machine Learning library for Python designed to work with NumPy and SciPy libraries.

Furthermore, matRadiomics provides a range of supervised and unsupervised algorithms for statistical modeling and Machine Learning, such as SVM [13], Logistic Regression [21], Bayesian classifier [22], KMeans [23], and DBSCAN [24].

The application is supported on macOS, Windows, and Linux operating systems and it is distributed in both compiled (standalone MATLAB application) and non-compiled versions. The compiled version does not require a MATLAB license, but relies only on a MATLAB runtime. This characteristic makes matRadiomics well suited for application in clinical settings.

MatRadiomics also integrates ComBat [25], a data harmonization technique used to remove non-biological sources of variance in multisite studies.

In the first version of matRadiomics, ad hoc functionalities are grouped into three modules. The *dicomModule* includes two functions: one is used to parse and store all DICOM attribute names, tags, and value representations (VR) types in lists, and the other is used to obtain DICOM attribute values (e.g., the position of the slice, rescale intercept, and rescale slope) needed for other operations.

The *pyradiomicsModule* consists of a single function that configures the pyradiomics extractor with the settings chosen by the user.

The *classificationModule* consists of as many functions as the number of implemented classifiers in matRadiomics. It is used to execute model training, cross-validation, and to obtain the model performance metrics.

In this version of matRadiomics, we introduced the *NIfTIModule.*

The NIfTI (Neuroimaging Informatics Technology Initiative) is the most widely used data format for storing functional Magnetic Resonance Imaging (fMRI) because it solves the problem of spatial localization [26].

The decision to incorporate a function that allows importing images in NIfTI format was primarily driven by the necessity to import neuroimaging images that the Freesurfer software 7.3.2 [27] releases in nii format after the segmentation process.

*NIfTIModule* is an I/O module that allows the user to start a neuroimaging radiomics pipeline based on the NIfTI only. *NIfTIModule* has significantly broadened matRadiomics capabilities, allowing its utilization in Neuroimaging, whereas previously it was confined to support only images in DICOM format.

Furthermore, the *classificationModule* was expanded by incorporating additional functionalities, allowing users to configure the construction of two new predictive models directly from the application. These newly added models include SVM [12] and Random Forest [28], providing users with more options for building predictive models tailored to their specific needs.

### 2.2. Image Visualization

Starting a study in matRadiomics requires the creation of a new series. This allows us to set a folder where the results of extraction, selection, and Machine Learning processes are saved.

A series denotes the type of study that will be performed, so it is recommended to name the series descriptively.

Besides importing DICOM files, which were already enabled in the previous version of the matRadiomics application, it is now possible to import files in NIfTI format.

For neuroimaging studies in matRadiomics, we managed the import and reading of NIfTI images through a function that extracts the metadata and converts the volume into a matrix, a data structure that can be conveniently managed by the Matlab syntax.

Following the import procedure, volume normalization is also performed to standardize medical images, enhance contrast, and reduce noise.

The normalization process is of paramount importance for the following:

- Standardizing medical images to facilitate the analysis and comparison of images from different sources or acquired at different times, as they are often acquired using different devices or protocols, resulting in variations in intensity levels and grayscale;
- Enhancing contrast by redistributing intensities to better highlight important details;
- Preparing for feature extraction by reducing the impact of unwanted variations in value scales. This can make the results more consistent and facilitate the extraction of relevant information;
- Reducing noise to improve image quality.

### 2.3. Segmentation

Segmentation can be achieved through several methods:

1. Manual Segmentation: In this approach, the target region is manually delineated through user interaction. This method is time-consuming and prone to operator-dependent variability;
2. Semi-automatic Segmentation: Algorithms such as Region Growing or Thresholding are employed, utilizing similar criteria to delineate the target. However, these methods often necessitate manual refinement of the generated masks, introducing potential operator-dependent errors;
3. Automatic Segmentation: Deep Learning techniques and pre-trained algorithms are utilized to automatically segment specific regions of interest. This approach minimizes operator-dependent errors, although it requires substantial amounts of labeled data for training and significant computational resources for execution.

In matRadiomics, both manual and semi-automatic segmentation algorithms are employed to delineate the target region of interest.

Although these two types of segmentation have also been enabled on NIfTI format images in matRadiomics, it is common practice to use automatic segmentations for neuroimaging with Freesurfer software representing a widely recognized gold standard in this field.

Freesurfer excels in segmenting several brain tissues, including gray matter, white matter, cerebrospinal fluid, and subcortical structures like the thalamus. Moreover, it reliably identifies intricate structures such as the corpus callosum and the mesencephalic bridge.

The segmentation outputs generated by Freesurfer are typically in NIfTI format, which is now compatible with the new matRadiomics extension.

The ability to import neuroimaging data generated by Freesurfer allows the user to proceed with subsequent steps of radiomics analysis within matRadiomics.

### 2.4. Extraction, Harmonization and Selection of Radiomics Features

MatRadiomics closely follows the guidelines of the reference manual “Image Biomarker Standardization Initiative” [29] for the standardization and interpretation of images used in medical research.

To proceed with feature extraction in the application, PyRadiomics is already set as the default extractor. When the PyRadiomics extractor option is enabled, it handles preprocessing tasks such as resampling, resegmentation, and discretization. However, if matRadiomics is used for neuroimaging, and the imported segmentations are obtained through the segmentation process performed with Freesurfer, the preprocessing methods in the application remain disabled because the imported masks are already preprocessed.

Features harmonization, carried out in the case of multi-centric studies, is managed by the ComBat package [25].

Furthermore, in addition to the existing feature selection methods in matRadiomics, a new feature selection method has been integrated: feature selection using the Random Forest model [30].

The feature selection process using the Random Forest model involves creating a set of decision trees, known as “random decision trees”. Each tree is trained on a random subset of the training data and utilizes a random subset of the available features.

After training, the model calculates the importance of each feature in the dataset by evaluating its contribution to improving the accuracy of decision tree predictions. Features that significantly enhance accuracy are considered more important.

With the features found to be significant during the selection process, it is possible to proceed with the building of a predictive model.

### 2.5. Machine Learning

The *classificationModule* is used to create a predictive model. This module is based on SciKit-learn [11], an open-source Machine Learning library that provides a wide range of tools for several Machine Learning tasks, including classification, regression, clustering, dimensionality reduction, model selection, and data preprocessing.

Within this module, in addition to the Machine Learning methods natively implemented in matRadiomics such as LDA [15], KNN [14], and SVM [13], the Random Forest method [30] was added.

Upon selecting the classification model, the dataset is partitioned into training and test sets, and the user can specify the size of both sets directly from the matRadiomics GUI.

These classification models yield accuracy, precision (True-negative Rate), and recall (True-positive Rate), which are then displayed in the application. The advantage of this module lies in its ability to empower clinicians to build different prediction models effortlessly with an iterative approach while obtaining performance metrics from these models. With these parameters, users can directly access the confusion matrix and the ROC curve plots.

## 3. Case Study

**Figure 1:**
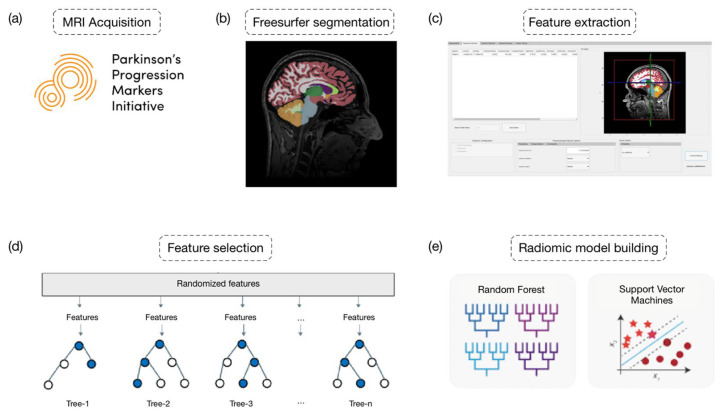
Radiomics workflow. In detail: (**a**) MRI acquisition; (**b**) Freesurfer segmentation; (**c**) feature extraction; (**d**) feature selection; (**e**) radiomic model building.

To assess the reliability and robustness of our matRadiomics extension, we conducted the radiomics workflow within the matRadiomics environment, utilizing openly available data from the Parkinson’s Progression Markers Initiative (PPMI) dataset (https://www.ppmi-info.org/accessdata-specimens/download-data (accessed on 17 February 2024)), as shown in Figure 1a.

The PPMI is a longitudinal, multi-center study aimed to identify biomarkers for Parkinson’s disease progression. It involves the collection of several data types, including clinical, imaging, genetic, and biochemical data, from both Parkinson’s patients and healthy controls over an extended period.

Specifically, we included 109 patients with Parkinson’s disease at baseline along with 118 healthy controls, matched for age and sex, all of whom underwent MRI imaging.

The MRI image segmentation was conducted using Freesurfer 7.3.2 and its standard recon-all command pipeline, as illustrated in Figure 1b. Within the Freesurfer output folder, the mri directory contains the aseg file, which includes masks of brain areas reconstructed by Freesurfer.

**Figure 2:**
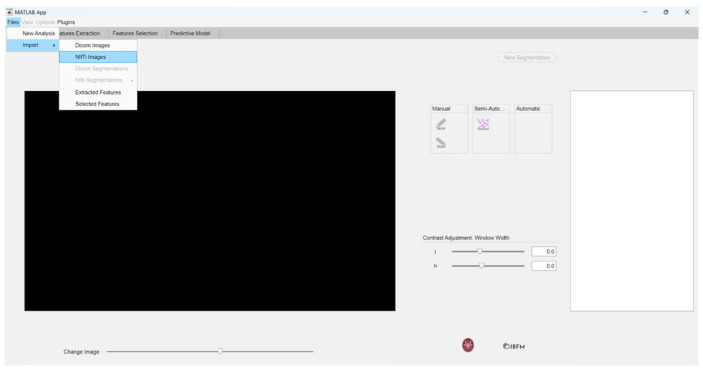
Importing NIfTI images.

We started our case study by initially creating a new series. We first loaded NIfTI images into matRadiomics by clicking the following buttons: *Files*, *Import*, and *NIfTI images*, as illustrated in Figure 2.

**Figure 3:**
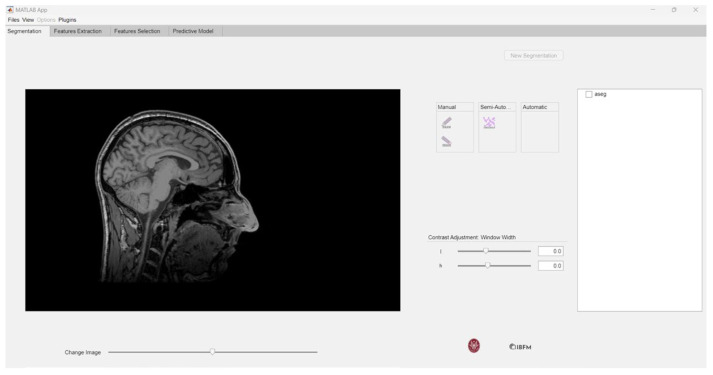
Importing NIfTI segmentations.

This process opens a window allowing the user to import the segmentation corresponding to the previously loaded MRI image in NIfTI format, as depicted in Figure 3.

**Figure 4:**
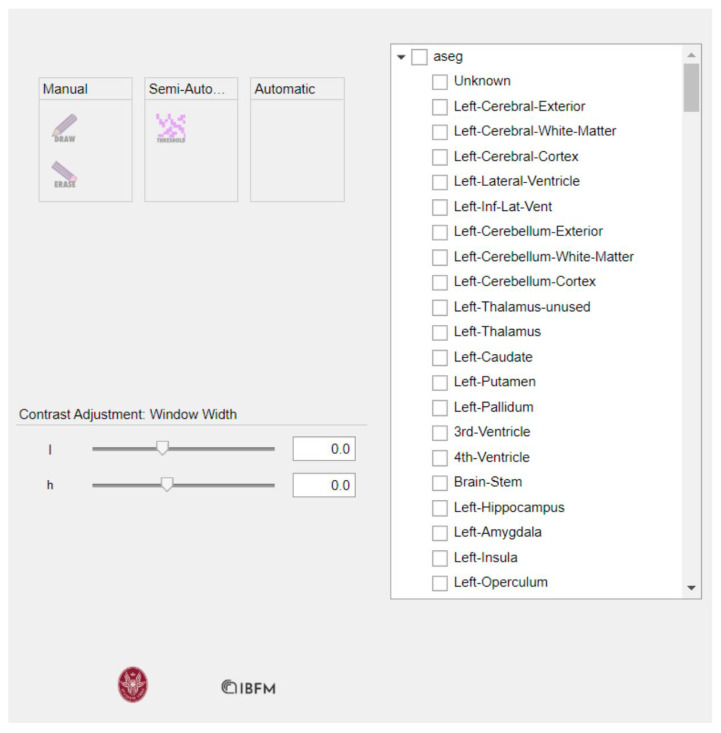
Checkbox containing the labels.

The segmentation import function generates a tree structure, where each leaf is linked to the labels of the imported segmentation. This tree is invoked in the callback function responsible for handling the use of the check box on the right side of the interface, as shown in Figure 4.

**Figure 5:**
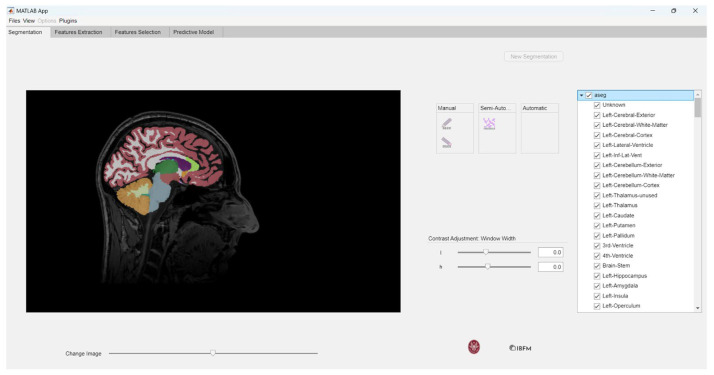
Overlay of the masks on the MRI images.

Within the checkbox, users have the option to select the aseg box, which overlays all segmentation labels on the image, as demonstrated in Figure 5. We examined the overlays of our masks on the MRI images to validate the accuracy of the Freesurfer segmentation.

**Figure 6:**
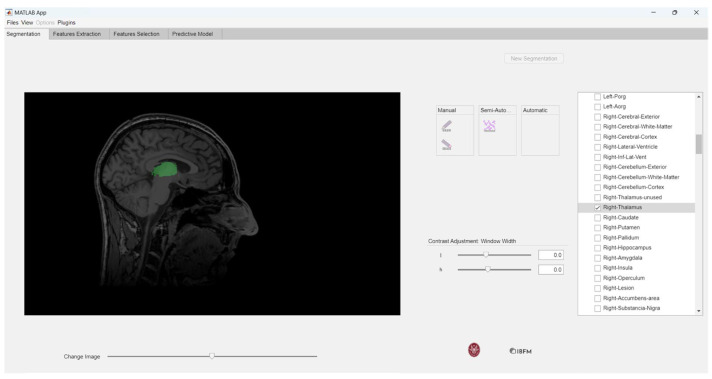
Selection of the mask of interest (right thalamus).

Alternatively, users can choose to check the boxes corresponding only to the masks of interest, as depicted in Figure 6.

The ability to selectively choose masks of interest is crucial in neuroimaging studies because researchers often need to focus on specific brain regions identified as target areas. Once the desired labels have been selected, the extraction of features can proceed, as shown in Figure 1c.

Feature extraction using PyRadiomics is supported in matRadiomics through the Python module *pyradiomicsModule*, which is managed by a flag within the same module, wherein different extraction functions are implemented. This flag enables the user to switch between extraction functions tailored for different image formats.

The feature extraction process involves the following steps:

1. Invoking the *getFeatures* function from MATLAB and supplying the necessary data for extraction;
2. Configuring the extractor parameters;
3. Initializing the extractor;
4. Performing feature extraction;
5. Sending the data back to MATLAB;
6. Converting the data into standard MATLAB variables;
7. Visualizing and saving the extracted features.

**Figure 7:**
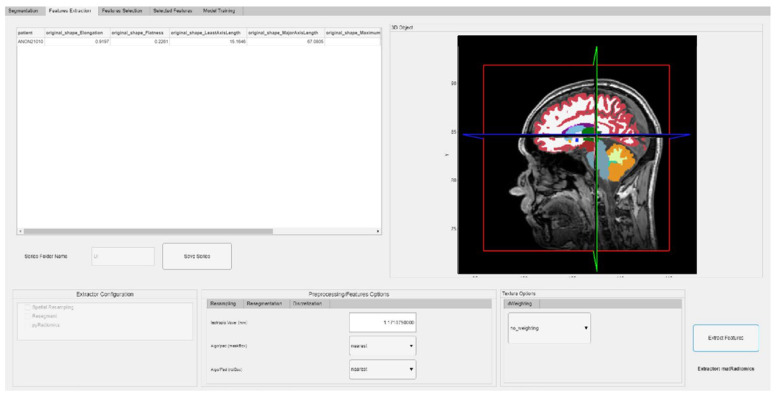
Feature extraction.

To execute these steps, users can simply click the Extract Features button, which is linked to the callback function *ExtractFeaturesButtonPushed*. The extracted features will then be displayed in the Feature Extraction tab, as illustrated in Figure 7.

The extracted features are saved in a CSV file in the Extracted Features folder, which is located within the previously created series folder. Additionally, within this folder, a file named *current_app_version* is saved. This file contains information about the application version, the used extractor, and the extraction options for that specific series.

We extracted 290 features using PyRadiomics default settings, and through the matRadiomics user interface, we labeled each healthy control as 0 and each PD patient as 1.

Due to the large number of available features, it was necessary to proceed with their selection as shown in Figure 1d, as redundant features could increase the complexity of the learning model and potentially lead to overfitting.

Depending on the type of algorithm used for feature selection, the outcome can be a subset of features or a score assigned to each feature. For feature selection, the Random Forest method from the Feature Selection tab was chosen.

The selected features were used to train two classification models: SVM and Random Forest. The dataset was split into a training set and test sets, with a specified test size of 20%, directly from the matRadiomics GUI.

To improve model performance and prevent over and under-fitting, hyperparameter tuning using randomized search was performed.

Randomized search was chosen as the tuning technique because it randomly explores a subset of the hyperparameter space, without examining all possible combinations, thus reducing the computational cost. A 5-fold stratified cross-validation and 10 repetitions to evaluate the model’s performance were set up.

The performance metrics obtained from the two trained models after hyperparameter tuning are reported in Table 1.

These performance metrics provide insight into how well the classification models are able to distinguish between healthy controls and Parkinson’s disease patients.

In this context, accuracy represents the proportion of correctly classified instances out of the total instances, while precision measures the proportion of true positive instances among all instances classified as positive. Recall, also known as sensitivity, measures the proportion of true positive instances that were correctly identified by the model out of all actual positive instances.

These results suggest that both SVM and Random Forest models perform reasonably well in discriminating between controls and Parkinson’s disease patients, with Random Forest exhibiting slightly higher accuracy compared to SVM.

## 4. Discussion

While several software tools exist for conducting radiomic analyses [31], they often come with inherent limitations that can hinder their utility in clinical practice. One significant drawback is the fragmented nature of traditional radiomics pipelines, which typically involve the use of multiple software platforms for image importation, segmentation, feature extraction, and model construction. This disjointed workflow not only complicates the process, but also increases the likelihood of errors and inconsistencies due to data transfer between different software environments.

Moreover, many existing toolboxes lack user-friendly interfaces and comprehensive functionality, making them inaccessible to clinicians and researchers without extensive programming expertise. This complexity can deter adoption and limit the widespread application of radiomics in clinical settings, where efficient and intuitive tools are essential for seamless integration into routine practice.

Furthermore, the compatibility of existing toolboxes with different image formats and segmentation algorithms is often limited, restricting their applicability to specific imaging modalities or segmentation techniques. This constraint poses challenges for researchers working with diverse datasets and necessitates manual intervention to adapt existing workflows to new data types or processing methods.

The integration of matRadiomics into neuroimaging studies represents a significant advancement in the field of medical image analysis. This extension enables researchers and clinicians to seamlessly conduct radiomic analyses on neuroimaging data, especially using brain segmentations made with the Freesurfer software.

One of the key strengths of matRadiomics lies in its ability to handle different image formats, including DICOM and NIfTI, thereby facilitating the analysis of several types of medical images commonly used in neuroimaging research. By incorporating features such as image import, segmentation, feature extraction, and Machine Learning within a single interface, matRadiomics streamlines the radiomics workflow, making it more efficient and user-friendly.

In addition, the way matRadiomics seamlessly interacts with the Python environment, which leverages Matlab’s official support, contributes to the ease of maintenance and extension of matRadiomics with new functionalities. This design choice streamlines the development process and facilitates the incorporation of updates or enhancements, ensuring the continued evolution of matRadiomics to meet the evolving needs of users in medical image analysis.

The case study conducted using data from the PPMI dataset demonstrated the practical utility of matRadiomics in neuroimaging research. By leveraging Freesurfer segmentation and PyRadiomics feature extraction, the application enabled the extraction of a comprehensive set of radiomic features from MRI images of both healthy controls and Parkinson’s disease patients. The subsequent application of Machine Learning algorithms, such as SVM and Random Forest, facilitated the construction of predictive models to distinguish between the two groups with reasonable accuracy.

The performance metrics obtained from the trained models indicate the potential of radiomics in aiding the diagnosis and characterization of neurological disorders like Parkinson’s disease. The high accuracy, precision, and recall values suggest that the extracted radiomic features capture meaningful information relevant to disease status, highlighting the utility of matRadiomics in clinical decision making.

Furthermore, the seamless integration of Freesurfer segmentation into the matRadiomics workflow enhances its applicability in neuroimaging research. By allowing users to import and utilize Freesurfer-generated segmentations directly within the application, matRadiomics expands its functionality to encompass the analysis of complex brain structures with minimal manual intervention.

## 5. Conclusions

In conclusion, the matRadiomics extension emerges as a valuable tool for researchers and clinicians involved in neuroimaging studies. Its comprehensive feature set, combined with its user-friendly interface and compatibility with popular neuroimaging software like Freesurfer, establishes matRadiomics as a versatile platform for conducting radiomic analyses in the context of neurological disorders.

In recognizing the promising capabilities of matRadiomics in neuroimaging research, it is essential to recognize the inherent limitations and potential biases that may be of influence, thus strengthening the generalizability and robustness of radiomics analyses conducted with matRadiomics in the interpretation of results. Since our analysis is mainly based on a single case study oriented towards evaluating the functioning of matRadiomics, there is a risk of selection errors as the characteristics of the subject included may not fully represent the broader population of interest. Furthermore, the impact of imaging parameters on feature extraction cannot be underestimated, as variations in acquisition protocols and equipment settings can introduce variability in radiomics features. In future investigations, we will seek to incorporate different cohorts and standardize imaging protocols to mitigate these potential sources of bias.

To further enrich the potential of matRadiomics in neuroimaging research, the next advances could address several aspects.

The process of handling large amounts of neuroimaging data could be improved by introducing batch processing capabilities and optimizing computational resources.

Moreover, incorporating cloud computing solutions could provide scalability and flexibility, enabling researchers to analyze data remotely and collaborate across different institutions.

We will proceed with the addition of new segmentation algorithms within matRadiomics by inserting cutting-edge Deep Learning methods.

Furthermore, large validation studies could be performed to assess the reproducibility and clinical relevance of radiomics features extracted via matRadiomics in different cohorts and imaging protocols.

Finally, to ensure efficient processing of T1-weighted volumes for radiomic analysis, a future development involves executing a full recon-all FreeSurfer operation on each volume within an isolated Docker container. This approach is expected to optimize runtime, with typical execution times ranging from 3 to 4 h per volume, depending on hardware specifications. This process will be initiated directly from MATLAB, detached from the original system call, enabling seamless interaction with matRadiomics. Utilization of the official FreeSurfer Docker image (https://hub.docker.com/r/freesurfer/freesurfer/ (accessed on 18 February 2024) will also facilitate compatibility with Windows operating systems.

The approach that we aim to utilize for integrating Freesurfer into matRadiomics represents a replicable and extensible model for interacting with other segmentation tools that employ advanced technologies, such as Deep Learning algorithms or advanced segmentation algorithms, even outside the context of neuroimaging. This flexibility paves the way for potential software development, allowing matRadiomics to be easily adapted for the analysis of a wide range of medical imaging data.

By focusing on these aspects, matRadiomics holds promise in advancing as a valuable tool for enhancing our comprehension of neurological disorders and improving patient care within clinical environments.

## Figures and Tables

**Table 1:** Performance metrics of SVM and Random Forest models.

	RF ^1^	SVM ^2^
Accuracy	0.85	0.81
Precision	0.84	0.86
Recall	0.92	0.83

^1^ Random Forest, ^2^ Support Vector Machine.

## Data Availability

Source code, documentation, and examples are available upon request to the authors. After the publication of this article, the software will be available on the institutional websites of the authors (IBFM-CNR and Sapienza University). Data used in the preparation of this article were obtained from the Parkinson’s Progression Markers Initiative (PPMI) database (https://www.ppmi-info.org/access-data-specimens/download-data (accessed on 17 February 2024). For up-to-date information on this study, visit https://www.ppmi-info.org (accessed on 17 February 2024).
